# Unraveling the Fungal Community Dynamics in Heat-Tolerant Coral *Turbinaria* sp. During Bleaching in South China Sea

**DOI:** 10.3390/jof11120832

**Published:** 2025-11-25

**Authors:** Xinye Chen, Xinyu Liao, Li Mo, Xumeng Ren, Yaozu Li, Qili Hou, Simon Wing-Fai Mok, Riming Huang, Jijia Sun, Xiaoyong Zhang

**Affiliations:** 1 University Joint Laboratory of Guangdong Province, Hong Kong and Macao Region on Marine Bioresource Conservation and Exploitation, College of Marine Sciences, South China Agricultural University, Guangzhou 510642, China; xinyeeast@gmail.com (X.C.); liaoxinyu2023@163.com (X.L.); molly5792@163.com (L.M.); renxumeng22@mails.ucas.ac.cn (X.R.); liyaozu17@163.com (Y.L.); kili27532016@gmail.com (Q.H.); 2 State Key Laboratory of Quality Research in Chinese Medicine, Macau University of Science and Technology, Macao, China; smok625@gmail.com; 3 Guangdong Provincial Key Laboratory of Nutraceuticals and Functional Foods, College of Food Science, South China Agricultural University, Guangzhou 510642, China; huangriming@scau.edu.cn

**Keywords:** coral reef, coral bleaching, South China Sea, coral-associated fungi

## Abstract

Coral bleaching is a multifactorial stress response in which the breakdown of symbiosis with algal and bacterial partners has been well characterized, but the role of fungal communities remains largely unexplored. Here, we tracked the temporal dynamics of coral-associated fungi in *Turbinaria* sp. across three defined bleaching stages under natural thermal stress. In total, 161 genera from six phyla were detected. From the unbleached to partly bleached stage, fungal Simpson diversity declined, whereas observed richness slightly increased; putative pathogenic genera (e.g., *Apiotrichum*, *Curvularia*, *Exserohilum*, and *Schizophyllum*) rose sharply (39.44%→69.04%), whereas parasitic fungi decreased (33.01%→11.72%). From the partly to fully bleached stage, diversity rebounded. Co-occurrence networks became more complex initially (nodes 86→98; edges 454→809; average degree 10.56→16.51) but then collapsed below baseline (nodes 98→65; edges 809→196; average degree 16.51→6.03), indicating stress-driven restructuring. The proportion of positive correlations declined steadily (98.68%→93.82%→77.55%), suggesting a shift toward more competitive and unstable community structures under stress. Our findings demonstrate that fungal communities actively respond to thermal stress and exhibit distinct compositional and ecological shifts during bleaching, pointing to their overlooked but potentially significant role in coral health and deterioration. This study highlights the need to integrate fungal dynamics into the broader understanding of holobiont responses to coral bleaching.

## 1. Introduction

Coral bleaching results from the loss of symbiotic dinoflagellates (Symbiodiniaceae), producing the visible disappearance of algal pigments. While elevated sea temperatures are the principal trigger of mass bleaching [1], recent work has clarified the cellular pathways that mediate this response. Environmental stressors, including high irradiance, thermal anomalies, salinity fluctuations, inorganic nutrient enrichment, trace metals, and shifts in the nitrogen-to-phosphate ratio, place corals under suboptimal conditions which induce excessive accumulation of reactive oxygen and nitrogen species (ROS/RNS) in host and symbiont cells. This oxidative and nitrosative stress destabilizes the cnidarian–algal symbiosis and activates host defense programs such as symbiont expulsion and xenophagy that remove algal endosymbionts from the coral host [2].

Remarkably, some coral reefs have demonstrated enhanced thermal tolerance in response to recurrent marine heatwaves [3]. This resilience reflects adaptation of corals to external environments, including symbiont shuffling/switching within Symbiodiniaceae, host genetic adaptation, and physiological acclimatization [4]. Multiple studies show that heat-tolerant corals such as *Platygyra* sp. and *Favites* sp. can increase thermal tolerance by shifting from heat-sensitive algal endosymbionts to more tolerant lineages such as *Durusdinium* [5]. In addition, coral-associated bacteria also contribute to thermal resilience [6]. The bacterial microbiome is far more diverse and abundant than algal endosymbionts and underpins host carbon, nitrogen, and sulfur cycling [7]. During bleaching, bacterial communities often shift toward opportunistic and pathogenic heterotrophs with concurrent declines in putatively beneficial taxa [8]. Selected “beneficial microorganisms for corals” (BMCs) have been proposed as probiotics; experimental inoculations can enhance host heat tolerance, suggesting a promising but still developing strategy to mitigate bleaching.

As another key component of the coral holobiont, fungi have received far less attention than coral-associated bacteria and algal endosymbionts [9]. Historically, coral-associated fungi were mainly regarded as pathogens, implicated in diseases such as skeletal eroding band, dark spot syndrome, and sea-fan aspergillosis [10,11,12]. However, their potential roles in coral bleaching remain poorly characterized. Previous studies have suggested that elevated seawater temperatures approaching 30 °C, which is near the optimal growth range for many fungi, may enhance fungal metabolic activity and virulence, potentially compromising host immunity and increasing the risk of opportunistic infections [13]. For instance, although thermal stress (31.5 °C) was shown to induce a 176% increase in host-derived antifungal activity, it also accelerated fungal growth, thereby providing a temporal window for pathogen establishment before effective host defense could occur. In addition, as fungi are known to act as dominant pathogens of algae, their increased abundance under thermal stress may disrupt the mutualistic balance between coral hosts and their Symbiodiniaceae symbionts, potentially leading to abnormal nutrient cycling and holobiont destabilization [14]. These findings highlight the need to better understand the dynamics and functional roles of coral-associated fungi during bleaching events.

Coral bleaching, characterized by the breakdown of the symbiosis between corals and their endosymbiotic algae, results in a severe decline in photosynthetically derived nutrients and energy, leading to physiological stress and ultimately starvation [15]. While fungi cannot replace the trophic functions of algal symbionts, they represent a diverse and metabolically versatile component of the coral holobiont [16]. Fungi are known to produce antimicrobial and antioxidant compounds, modulate microbial interactions, and colonize coral tissues as either mutualists or pathogens [17,18]. While bleaching often reduces host immune capacity and coincides with opportunistic infections, antimicrobial metabolites from coral-associated fungi can suppress opportunists and may bolster host resistance [19]. Moreover, since the overproduction of ROS/RNS in coral cells is a major driver of oxidative stress and a primary cause of bleaching, the antioxidant capacity of certain coral-associated fungi merits attention. Several fungal taxa isolated from corals have been shown to produce potent antioxidant metabolites in vitro [20], suggesting a potential role in modulating host oxidative stress responses and possibly mitigating bleaching susceptibility.

While bleaching disrupts the stability and composition of the coral holobiont, including its bacterial and algal partners, similar functional shifts in fungal communities remain underexplored. In bacterial communities, consistent alterations in taxonomic and functional profiles have been characterized using 16S rRNA high-throughput sequencing [21]. Elevated seawater temperatures not only accelerate coral bleaching but also selectively favor the proliferation of potentially pathogenic bacterial taxa, while concurrently suppressing the growth of bacterial groups associated with probiotic or beneficial functions. Applying comparable multi-omic approaches to fungal symbionts may provide deeper insights into their ecological contributions. Prior studies have also profiled fungal communities in healthy versus bleached corals across bleaching events using high-throughput sequencing [22,23,24]. However, a binary focus on “healthy” and “bleached” overlooks community dynamics at intermediate stages. Time-resolved field sampling across defined bleaching stages is thus essential for a comprehensive analysis of fungal roles in bleaching.

*Turbinaria* sp. are widely distributed in the South China Sea and exhibit comparatively high thermal tolerance among scleractinians [25]. However, fungal ecological roles during bleaching in coral *Turbinaria* sp. remain largely unexplored. Investigating the dynamic responses of fungal communities under thermal stress may reveal stage-specific shifts in community composition, functional capacity, and interspecific interactions that either buffer or exacerbate bleaching outcomes. Therefore, this study aims to characterize the temporal trajectories of coral-associated fungal diversity, guild structure, and co-occurrence patterns across a natural bleaching event to better understand their potential contributions to coral health and dysbiosis under climate stress.

## 2. Materials and Methods

### 2.1. Study Sites and Sample Collection

**Figure 1:**
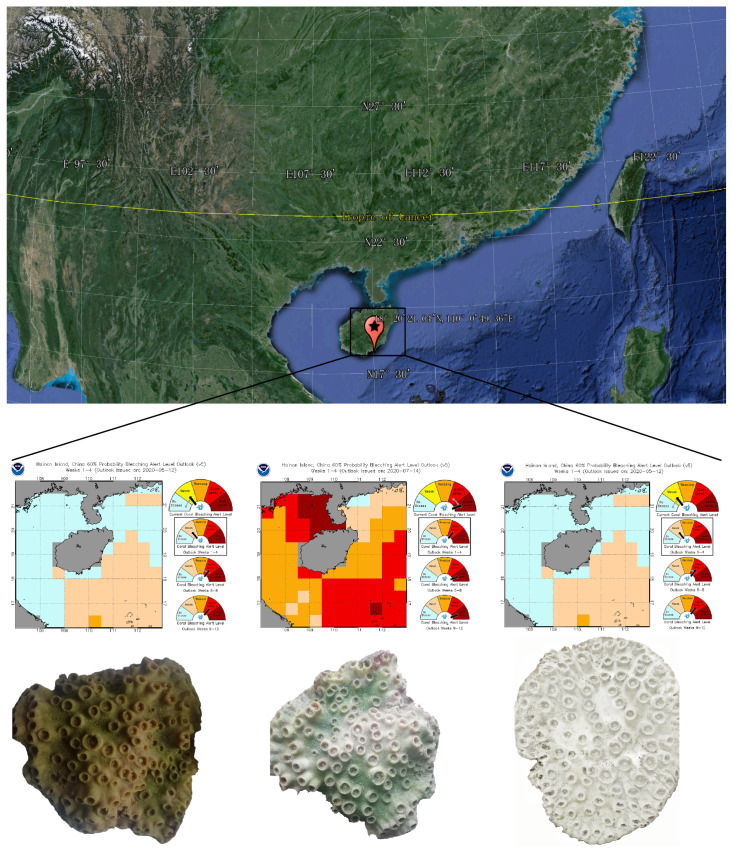
Sampling site, maps of heat stress and bleaching alert in May, July, and September 2020 (NOAA Coral Reef Watch, https://coralreefwatch.noaa.gov/product/vs/gauges/spratly_islands.php, accessed on 21 September 2020), and coal samples at different bleaching stages.

In this study, coral samples were collected from five *Turbinaria* sp. colonies that were repeatedly sampled across three bleaching stages, including unbleached (UT), partly bleached (PBT), and fully bleached (BT). Each colony was treated as a biological replicate, and temporal comparisons were performed within colonies to account for repeated measures, and statistical analyses were accordingly performed to account for non-independence of observations (Figure 1). Colony identity was confirmed using colony morphology and mitochondrial marker sequences. NOAA Coral Reef Watch satellite imagery was used for thermal stress monitoring and bleaching stage classification (NOAA Coral Reef Watch, www.coralreefwatch.noaa.gov, accessed on 21 September 2020). UT samples were collected in May 2020 during sustained positive sea surface temperature (SST) anomalies (daily SST: 27.13–30.55 °C), when the site was under NOAA Coral Reef Watch’s “Bleaching Watch” status and no visible bleaching was observed. PBT samples were collected in July 2020 (28.38–31.89 °C, “Alert Level 1”) as partial bleaching became apparent. BT samples were collected in September 2020 (29.01–31.34 °C, “Alert Level 2”) under elevated SST and cumulative heat stress conditions. At each time point, ~5 cm × 5 cm coral fragments were collected from each colony, gently rinsed twice with sterile 0.22 μm filtered seawater to remove loosely associated microbes, immediately flash-frozen in liquid nitrogen, and stored at −80 °C until DNA extraction. Sample collection procedures were approved by the Animal Ethics Committee of South China Agricultural University (approval ID: SYXK-2019-0136).

### 2.2. DNA Extraction and High-Throughput Sequencing

Genomic DNA was extracted using the PowerSoil DNA Isolation Kit (MO BIO Laboratories, Carlsbad, CA, USA) following the manufacturer’s protocol. The fungal ITS1 region was amplified with the universal primers ITS1F (5′-CTTGGTCATTTAGAGGAAGTAA-3′) and ITS2 (5′-GCTGCGTTCTTCATCGATGC-3′). PCR cycling comprised five steps: initial denaturation at 98 °C for 1 min; 30 cycles of 98 °C for 10 s, 50 °C for 30 s, and 72 °C for 30 s; and a final extension at 72 °C for 5 min. Reactions (15 µL) contained Phusion^®^ High-Fidelity PCR Master Mix (New England Biolabs, Ipswich, MA, USA), 0.2 µM of each primer, and 10 ng template DNA. Negative controls, including extraction blanks and no-template PCR reactions, were included throughout the workflow and sequenced in parallel. No amplification was detected in controls. ASVs observed in blanks were removed from the final dataset.

### 2.3. Sequence Analysis and Taxonomic and Functional Classification

Raw paired-end Illumina reads were processed in QIIME 2 (v2023.2) [26]. Sequences were demultiplexed and quality-filtered using the q2-demux plugin. Denoising was performed with DADA2 [27], which included merging of paired reads, removal of low-quality sequences, barcode assignment, and identification of chimeric reads (via the UCHIME algorithm). This yielded a high-resolution feature table of amplicon sequence variants (ASVs). ASVs were aligned using q2-alignment and a rooted phylogenetic tree was constructed via q2-phylogeny [28]. Taxonomic classification was conducted using the q2-feature-classifier plugin with the naïve Bayes classifier trained on the UNITE database (QIIME release for Fungi, v9.0) [29]. To ensure taxonomic specificity, non-fungal sequences (e.g., plant, metazoan, protist, or unassigned ASVs) were filtered out based on these assignments. Only ASVs confidently classified within the fungal kingdom (Fungi) were retained for community composition and functional analyses.

Functional annotation of fungal ASVs was conducted using the FUNGuild Python script (v1.1) [30] to infer putative ecological functions based on a curated database of fungal trophic modes and guilds. Only annotations with “Probable” or “Highly Probable” confidence rankings were retained for downstream analyses to ensure functional reliability.

### 2.4. Statistical Analysis

Between-sample community dissimilarity (β-diversity) was assessed using Aitchison distance in R (v4.3.1) with the vegan and ggplot2 packages, accounting for the compositional nature of sequencing data [31,32]. Univariate comparisons were conducted using parametric test (*t*-test) after normality assessment (Shapiro–Wilk test) [33]. For differential abundance analyses, *p*-values were adjusted using the Benjamini–Hochberg false discovery rate (FDR) procedure to correct for multiple hypothesis testing.

Co-occurrence analysis was based on pairwise Pearson correlations among genera; edges were retained at FDR-adjusted *p* < 0.05 (Benjamini–Hochberg) and |r| > 0.6. Network indices (average degree, density, and the proportion of positive vs. negative edges) were computed with igraph [34], and networks were exported to Gephi (https://gephi.org/) for visualization [35]. For each health status, a separate network was plotted with nodes representing fungal guilds and edges representing positive or negative associations.

## 3. Results

### 3.1. Fungal Community Diversity and Structure

**Figure 2:**
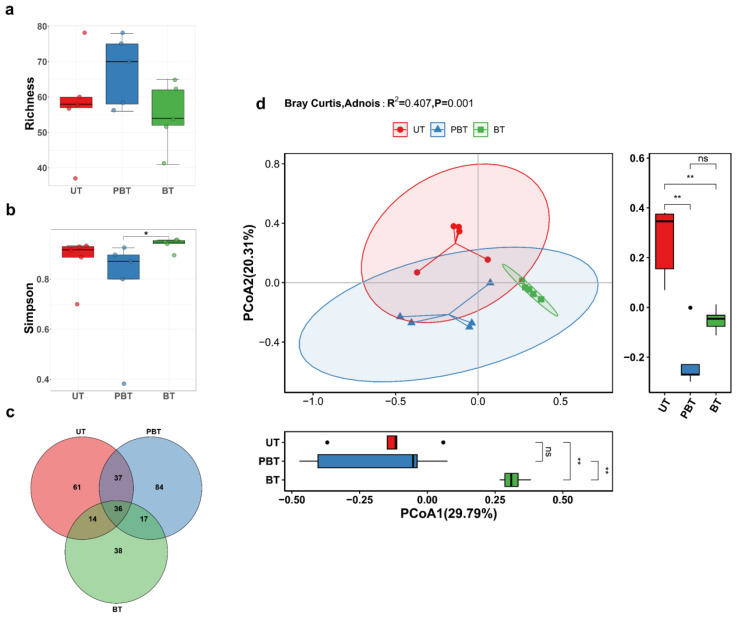
α-diversity index (**a**,**b**), Venn diagram (**c**), and Principal co-ordinates analysis (**d**) of fungal communities in coral *Turbinaria* sp. at different bleaching stages. Statistical significance: *p* < 0.05 (*), *p* < 0.01 (**); ns: not significant (*p* ≥ 0.05). UT: Unbleached *Turbinaria* sp., n = 5. PBT: Partly bleached *Turbinaria* sp., n = 5. BT: Bleached *Turbinaria* sp., n = 5.

A total of 15 *Turbinaria* sp. samples spanning three bleaching stages were collected from the South China Sea, and their fungal communities were profiled by high-throughput amplicon sequencing of the ITS1 rDNA region (Table S1). Sequence analysis yielded ~778,112 fungal reads after removal of non-fungal and low-quality sequences (mean 51,874 reads per sample), and 287 ASVs. The rarefaction curves for each sample plateaued as sequencing depth increased (Figure 2a), suggesting that the sequencing depth was sufficient to capture the fungal diversity in this study (Figure S1). α-Diversity varied across stages (Figure 2a,b; Table S2). Although no statistically significant stage-wise difference was detected for observed richness, the mean richness in the PBT was modestly higher (mean ± 95% CI: 67.4 ± 12.3) compared to both UT (58 ± 18.1) and BT (54.8 ± 11.7) stages. Conversely, Simpson diversity was significantly elevated in BT (0.94 ± 0.03) compared to PBT (0.78 ± 0.3; *p* = 0.032), with no marked difference between UT and BT. Venn diagrams showed shared and stage-exclusive fungal ASVs (Figure 2c). Fungal community composition differed significantly across bleaching stages (PERMANOVA, R^2^ = 0.407, *p* = 0.001), indicating that bleaching stage explained 40.7% of the variance in community structure (Figure 2d), indicating stage-structured turnover. Thirty-six ASVs were shared across all stages, and stage-exclusive ASVs were most numerous in PBT (84), followed by UT (61) and BT (38).

### 3.2. Diversity and Composition of Fungal Community

**Figure 3:**
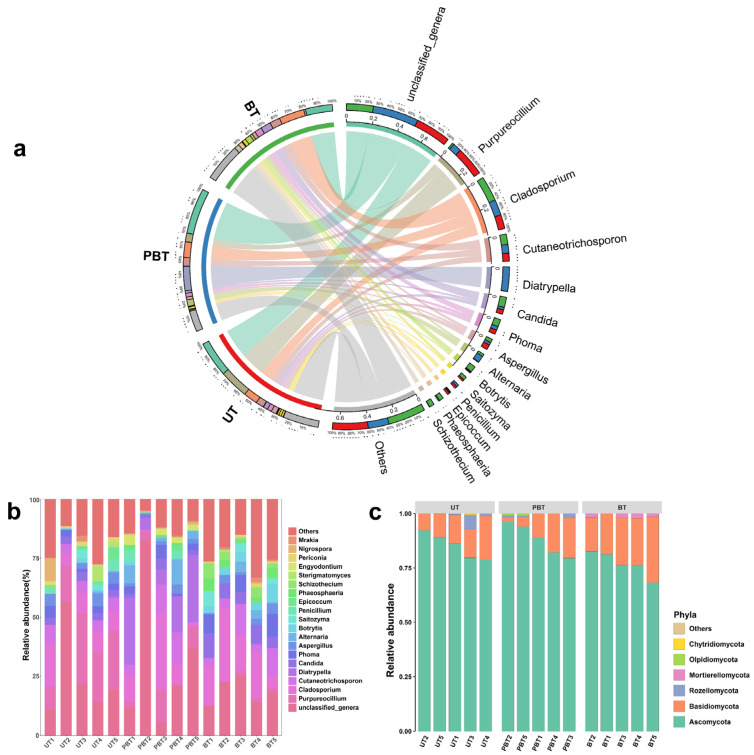
Relative abundance of fungi distributed in each sample at genus (**a**,**b**) and phylum (**c**) level in coral *Turbinaria* sp. UT: Unbleached *Turbinaria* sp., PBT: Partly bleached *Turbinaria* sp., BT: Bleached *Turbinaria* sp.

**Figure 4:**
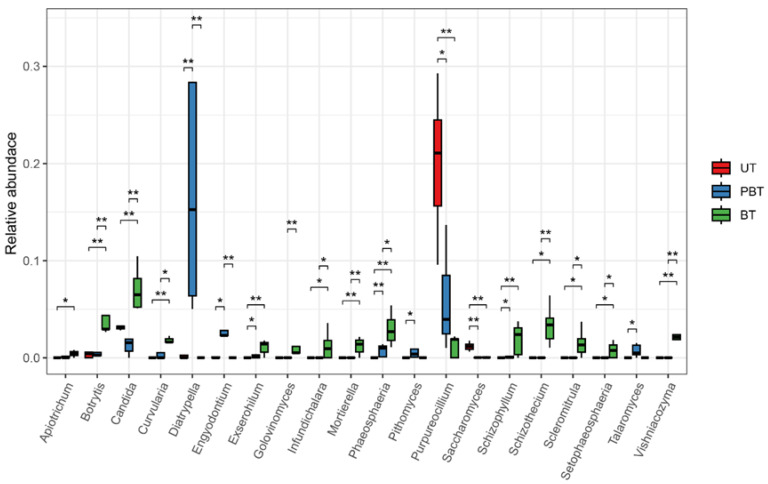
Relative abundance of significantly different (*p*-value) fungal genera among different coral samples. Statistical significance: *p* < 0.05 (*), *p* < 0.01 (**). UT: Unbleached *Turbinaria* sp., PBT: Partly bleached *Turbinaria* sp., BT: Bleached *Turbinaria* sp.

Taxonomic classification results showed that all 287 ASVs were assigned at the phylum level (Figure 3c), spanning *Ascomycota*, *Basidiomycota*, *Chytridiomycota*, *Mortierellomycota*, *Olpidiomycota*, and *Rozellomycota*. Across samples, *Ascomycota* (84.64%) and *Basidiomycota* (13.68%) accounted for the vast majority of reads. In total, 70.45% of reads were classified to 161 genera (Figure 3a), with the highest assignment rate in BT (81.45%) and the lowest in PBT (68.3%). Dominant genera varied across bleaching stages (Figure 3b, Table S3): at the UT stage, *Purpureocillium* (20.22%), *Cladosporium* (9.70%), and *Cutaneotrichosporon* (5.50%) were prevalent; in PBT, *Diatrypella* (16.75%), *Cladosporium* (10.80%), and *Cutaneotrichosporon* (6.06%) dominated; by the BT stage, communities were enriched in *Cladosporium* (17.5%), *Cutaneotrichosporon* (7.11%), and *Candida* (7.03%). Twenty genera differed significantly among stages (Welch’s *t*-test; Figure 4, Table S4), including *Diatrypella*, *Purpureocillium*, *Candida*, *Schizophyllum*, and *Phaeosphaeria*, many of which showed monotonic increases or decreases with bleaching progression.

### 3.3. Fungal Functional Prediction

**Figure 5:**
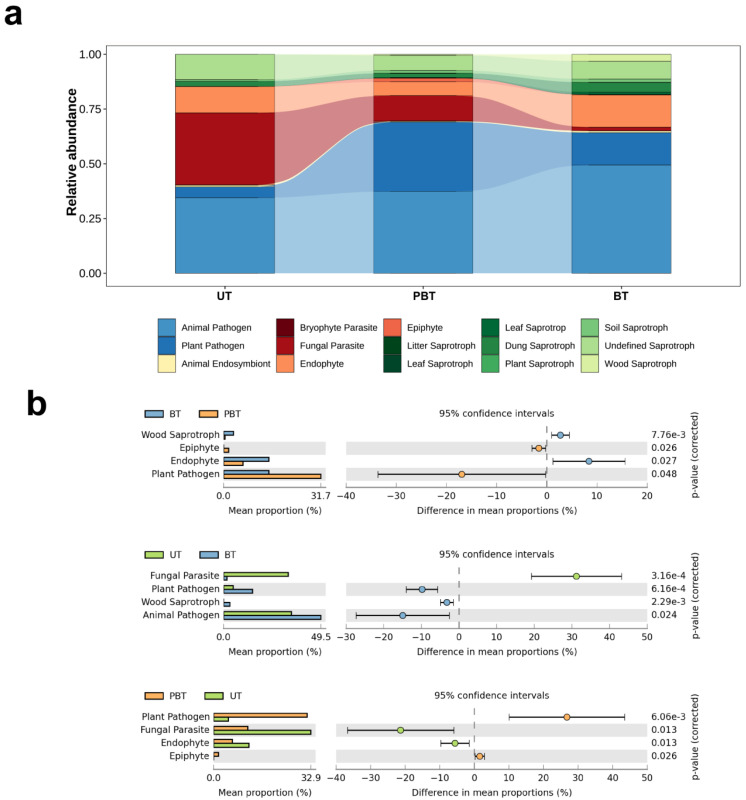
Relative abundance (**a**) and significant differences (**b**) of predicted fungal functional guilds in different bleaching coral *Turbinaria* sp. UT: Unbleached *Turbinaria* sp., PBT: Partly bleached *Turbinaria* sp., BT: Bleached *Turbinaria* sp.

The potential functional guilds of fungal communities at each bleaching stage were inferred with FUNGuild, providing a coarse-grained view of microecological responses to bleaching (Figure 5a). A total of 15 functional guilds were detected, dominated by pathogens, parasites, endophytes, epiphytes, and saprotrophs. To ensure interpretive reliability, only assignments with “Probable” or “Highly Probable” confidence rankings were retained for analysis. Functional annotations with lower confidence levels were excluded. Following this filtering step, 66.4% of the total fungal reads were retained for downstream guild-level interpretation. Notably, putative pathogenic fungi including both plant and animal pathogens were the dominant guild in PBT and BT, whereas parasites were most prevalent in UT. To investigate stage-wise shifts, differences in guild relative abundances were tested by *t*-tests (Figure 5b). In the first interval (UT→PBT), parasites and endophytes declined markedly, whereas pathogens increased markedly. In the second interval (PBT→BT), wood saprotrophs and endophytes rose substantially, accompanied by a pronounced decrease in plant pathogens.

### 3.4. Co-Occurrence Network Analysis of Fungal Communities

**Figure 6:**
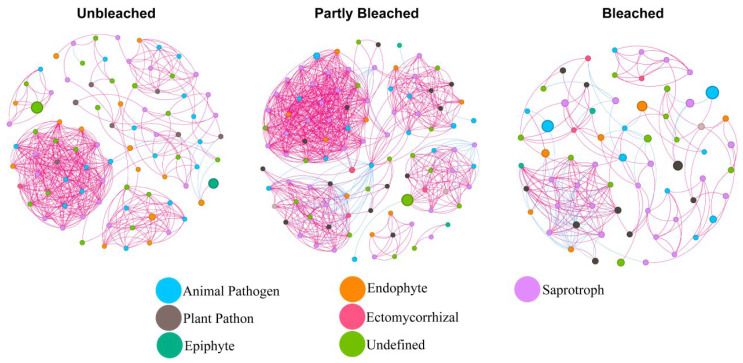
Network patterns of fungal interspecies interaction at functional group level in coral *Turbinaria* sp. at different bleaching stages. The colors of nodes in the networks represent different fungal guilds. Node sizes are scaled to reflect the relative abundance of each fungal taxon. Edges represent significant Spearman correlations (|r | > 0.6, *p* < 0.05). Red lines denote positive associations, while blue lines indicate negative associations between taxa.

The putative interspecific associations within the coral-associated fungal microbiome and their relationship to bleaching were examined using co-occurrence network analysis based on Pearson correlation coefficients (Figure 6). The topological characteristics of the co-occurrence networks revealed distinct differences among bleaching stages (Table S5). The number of nodes and edges, as well as the average degree, increased modestly in the first interval (nodes 86→98; edges 454→809; average degree 10.56→16.51), consistent with a transient rise in network connectivity. However, these topological characteristics declined below baseline in the next interval (nodes 98→65; edges 809→196; average degree 16.51→6.03), indicating network fragmentation under sustained heat stress. Notably, positive correlations exceeded negative correlations at all stages (positive 98.68%→93.82%→77.55%; negative 1.32%→6.18%→22.45%), with the UT sample hosting the highest proportion of positive correlations and the BT sample the lowest, suggesting a shift from predominantly positive toward increasingly negative or decoupled associations. Network modularity increased from UT (0.571) to BT (0.666), indicating stronger compartmentalization despite declines in overall density and degree.

## 4. Discussion

Coral-associated fungi are a major component of the coral holobiont, but their roles in coral bleaching remain unresolved [24]. Fungi are sensitive to warming [36], and sustained heat exposure during bleaching can restructure coral fungal communities. In addition, dysbiosis of coral *Symbiodiniaceae* can secondarily reshape the coral-associated fungal community [37]. However, direct tests of fungal–host interactions during bleaching are scarce. To address this gap, we profiled fungal diversity and community composition, inferred functional guilds, and analyzed co-occurrence network structure in *Turbinaria* sp. across three bleaching stages.

However, fungal community profiling in marine environments is subject to known methodological biases. The ITS1F/ITS2 primer pair preferentially amplifies *Ascomycota* and underrepresents *Basidiomycota* and early-diverging lineages (e.g., *Chytridiomycota*), potentially distorting observed community composition [38]. Moreover, amplicon-based sequencing using ITS primers in coral reef systems is inherently susceptible to non-fungal contamination. The ITS region is present in many eukaryotes, including algae, dinoflagellates, and metazoans; thus, non-fungal DNA may be co-amplified during PCR [39]. Although we implemented stringent taxonomic filtering to retain only ASVs confidently assigned to the fungal kingdom, it is possible that low-abundance or novel fungal lineages were inadvertently excluded due to ambiguous classification. These limitations highlight the need for more targeted primer sets, host-DNA depletion strategies, or shotgun metagenomic approaches in future studies to better capture the true diversity of coral-associated fungi.

The stage-structured changes in fungal diversity and abundance reflect community responses to rising thermal stress and bleaching (Figure 4). As the average SST increased in the first interval (Figure 1), the abundance of coral-associated fungal community rose modestly, whereas diversity declined slightly (Figure 2a,b). While heat stress can affect various physiological aspects of fungi such as spore germination, mycelial growth, and reproductive cycles [40], the coral-associated fungal community may transiently consolidate interactions and enrich thermotolerant or opportunistic taxa, consistent with probiotic/beneficial-microbe concepts [41]. Moreover, decline in zooxanthellae during bleaching can alleviate competition between fungi and zooxanthellae, potentially providing more favorable conditions for fungal aggregation. However, in the second interval, SST remained elevated and cumulative heat stress increased, accelerating bleaching progression. In this stage, the richness of the coral-associated fungal community decreased slightly, whereas diversity rebounded. As corals reached a fully bleached state, weakened host condition may have coincided with an increase in the relative abundance of putative pathogenic taxa [42], consistent with observed taxonomic shifts and altered co-occurrence network structure.

A total of 6 fungal phyla and 161 fungal genera were identified across all coral samples, indicating higher fungal diversity in *Turbinaria* sp. than previously reported (Figure 3b,c). While the dominant genera were broadly consistent across bleaching stages, their relative abundances varied substantially (Figure 3a). In UT, *Purpureocillium* was the most abundant genus. Although rarely reported from corals, this genus is known for its broad-spectrum antimicrobial activity and biocontrol potential [43]. Other dominant genera in unbleached samples, including *Cladosporium* and *Aspergillus*, are recognized producers of antioxidant secondary metabolites [44,45], which may help mitigate bleaching-associated oxidative stress by scavenging excess ROS [44]. In the context of bleaching, where ROS accumulation contributes to cellular damage and symbiont loss, such metabolites may play a protective role if fungi remain metabolically active within coral tissues. Although coral-associated probiotic fungi have not been validated in situ, such bioactive traits suggest potential roles in host resilience. In PBT, the dominant genus was *Diatrypella*, which is predominantly saprotrophic on angiosperm wood and also includes species recognized as plant pathogens in agricultural and forest ecosystems [46,47]. However, its functional role and potential pathogenicity in coral holobionts remain unclear. Additionally, although PBT showed no significant shift in observed richness, it exhibited the highest number of stage-exclusive ASVs yet the lowest genus-level assignment rate. This suggests that the community in this transitional bleaching stage may be enriched with rare or poorly characterized taxa that escape taxonomic classification based on current ITS reference databases. Such taxa may include opportunists or early responders to host stress, which could play unrecognized roles in coral holobiont reorganization during bleaching. The observed changes in fungal dominance across bleaching stages highlight the dynamic restructuring of fungal communities under thermal stress, with possible implications for host–microbe interactions and coral health.

The potential functional groups of fungi were inferred from taxonomic assignments using FUNGuild [48]. Guild-level predictions (Figure 5a,b) indicated a higher relative abundance of putative pathogenic taxa, such as *Apiotrichum*, *Curvularia*, *Exserohilum*, and *Schizophyllum* in PBT and BT samples compared to UT, suggesting the early emergence of taxa with pathogenic potential as bleaching progresses. This pattern may be associated with heat and oxidative stress-induced suppression of host immune defenses, which could increase coral susceptibility to opportunistic microorganisms. In parallel, the relative abundance of endophytic fungi declined during the initial thermal stress phase, while saprotrophic fungi increased, particularly in the BT stage. The loss of *Symbiodiniaceae* via autophagy-related mechanisms during bleaching may release cellular debris that serves as a substrate for saprotrophs [49]. Simultaneously, the reduction in photosynthate supply may compromise symbiotic associations with nutrient-dependent endophytes. These functional shifts suggest a transition in the ecological roles of coral-associated fungi across bleaching stages, possibly reflecting altered nutrient availability and host condition.

The co-occurrence networks revealed changes in fungal interaction architecture during bleaching, including a reduction in positive correlations (Figure 6). However, such correlation-based networks are exploratory and do not imply direct ecological interactions or causality [50]. During coral bleaching, elevated temperature can alter fungal physiology and community composition by suppressing heat-sensitive taxa, promoting thermotolerant species, and reshaping competitive dynamics for space and resources, ultimately leading to a restructured fungal community [51]. In addition, the loss of *Symbiodiniaceae* reduces photosynthate supply to the host, likely lowering nutrient availability to associated fungi [52]. This resource limitation may intensify competition and antagonistic associations, as reflected by the increased proportion of negative edges and the reduction in positive correlations observed in the co-occurrence network (Table S2).

Although the relative abundance of putative pathogenic taxa increased during the early bleaching stage, the fungal network exhibited higher connectivity and structural complexity, suggesting a transient consolidation of interspecific associations, potentially representing a short-term compensatory response to thermal stress [53]. This phase corresponded with increased network density and a modest rise in overall fungal abundance. In the later bleaching stage, fungal abundance declined and network complexity diminished, indicating the stress-induced collapse of microbial associations and reduced community responsiveness. These patterns are consistent with progressive immune impairment in the coral host and the onset of full bleaching.

## 5. Conclusions

In summary, this study reveals stage-specific shifts in coral-associated fungal diversity, composition, ecological guilds, and network structure across a bleaching event, highlighting the dynamic responses of fungal communities to thermal stress. In the initial interval, fungal diversity declined while richness increased slightly, accompanied by a transient consolidation of the co-occurrence network; in the later interval, diversity rebounded as networks became simplified and fragmented. Guild inference indicated an increase in the relative abundance of putative pathogenic fungi and a decrease in endophytic taxa during this period. As bleaching intensified and colonies became fully white, overall fungal abundance declined toward baseline, whereas diversity rebounded relative to the early stage. Concurrently, network complexity and connectivity decreased, consistent with coral dysbiosis. While these results delineate stage-structured shifts in fungal community in *Turbinaria* sp., several methodological limitations warrant caution in interpretation. Functional predictions based solely on ITS amplicon data and taxonomic inference through FUNGuild lack direct evidence of activity and may overlook uncharacterized or low-abundance taxa. Moreover, co-occurrence networks, while informative for community structure, remain correlative and cannot infer causality. Future studies should adopt complementary approaches such as meta-genomic or meta-transcriptomic analyses, targeted cultivation, and beneficial microbiome manipulation assays (e.g., co-cultivation and reinoculation), to better resolve the ecological roles of dominant fungal taxa in the context of coral bleaching.

## Data Availability

Raw sequencing data have been deposited in NCBI SRA under accession number PRJNA 1353113.

## Supplementary Materials

The following supporting information can be downloaded at: https://www.mdpi.com/article/10.3390/jof11120832/s1, Figure S1: Rarefaction curves of the Sobs index for coral samples at different bleaching stages; Table S1: Sampling information for different bleaching stages of corals; Table S2: Diversity index of fungal community in each coral sample; Table S3: Relative abundance of fungi distributed in each sample at genus level; Table S4: Adjusted *p*-values for significantly different genera for each coral sample; Table S5: Topological characteristics of the co-occurrence networks fungal interactions in different coral samples.
