# Supplementary material for: Unraveling the Fungal Community Dynamics in Heat-Tolerant Coral Turbinaria sp. During Bleaching in South China Sea
Source: J Fungi (Basel). 2025 Nov 25;11(12):832. doi: 10.3390/jof11120832 (PMC12733531; doi:10.3390/jof11120832)
Supplement: Supplementary file 1 [file jof-11-00832-s001.zip › jof-3926543-supplementary.pdf]

Table S1 sampling information for different bleaching stages of corals

sample ID	sampling date	bleaching stage	SST
UT1	2020.5	unbleached	29.21
UT2	2020.5	unbleached	29.21
UT3	2020.5	unbleached	29.21
UT4	2020.5	unbleached	29.21
UT5	2020.5	unbleached	29.21
PBT1	2020.7	partly bleached	31.85
PBT2	2020.7	partly bleached	31.85
PBT3	2020.7	partly bleached	31.85
PBT4	2020.7	partly bleached	31.85
PBT5	2020.7	partly bleached	31.85
BT1	2020.9	fully bleached	30.57
BT2	2020.9	fully bleached	30.57
BT3	2020.9	fully bleached	30.57
BT4	2020.9	fully bleached	30.57
BT5	2020.9	fully bleached	30.57

Table S2 Diversity index of fungal community in each coral sample

sample	UT1	UT2	UT3	UT4	UT5	PBT1	PBT2	PBT3	PBT4	PBT5	BT1	BT2	BT3	BT4	BT5
Shannon	2.947	2.159	3.400	3.558	3.467	2.702	1.693	2.969	2.745	2.905	1.691	2.293	2.571	2.318	2.165
Simpson	0.868	0.726	0.919	0.954	0.945	0.832	0.512	0.903	0.832	0.902	0.541	0.717	0.793	0.685	0.655
Richness	112	53	88	71	72	78	117	107	81	105	49	65	64	79	75
Chao1	113	58	89.5	71	72	81	118.6667	109	83	106.875	54	66	64	82.75	77
Ace	113.019	60.693	89.818	71.000	72.000	81.779	119.171	109.621	84.467	109.592	54.758	66.399	64.000	85.201	78.534
Sobs	112	53	88	71	72	78	117	107	81	105	49	65	64	79	75

Table S3 Relative abundance of fungi distributed in each sample at genus level

tax	UPD1	UPD2	UPD3	UPD4	UPD5	PBPD1	PBPD2	PBPD3	PBPD4	PBPD5	BPD1	BPD2	BPD3	BPD4	BPD5
unclassified_genera	0.110	0.563	0.217	0.142	0.195	0.119	0.825	0.057	0.207	0.375	0.126	0.228	0.253	0.140	0.181
Purpureocillium	0.097	0.155	0.301	0.212	0.246	0.025	0.040	0.138	0.010	0.085	0.000	0.000	0.022	0.020	0.019
Cladosporium	0.183	0.051	0.087	0.082	0.082	0.114	0.003	0.325	0.085	0.013	0.169	0.312	0.148	0.191	0.053
Cutaneotrichosporon	0.079	0.040	0.047	0.051	0.058	0.041	0.004	0.119	0.134	0.005	0.033	0.039	0.133	0.035	0.115
Diatrypella	0.000	0.000	0.003	0.000	0.008	0.285	0.050	0.064	0.154	0.285	0.000	0.000	0.000	0.000	0.000
Candida	0.028	0.032	0.038	0.029	0.027	0.016	0.007	0.047	0.000	0.019	0.105	0.065	0.051	0.082	0.048
Phoma	0.053	0.028	0.014	0.030	0.027	0.032	0.003	0.056	0.011	0.029	0.080	0.000	0.073	0.000	0.097
Aspergillus	0.049	0.000	0.024	0.054	0.049	0.013	0.009	0.017	0.039	0.021	0.005	0.035	0.012	0.026	0.044
Alternaria	0.000	0.003	0.000	0.009	0.005	0.076	0.000	0.013	0.106	0.033	0.000	0.000	0.044	0.037	0.005
Botrytis	0.000	0.004	0.019	0.006	0.000	0.015	0.002	0.002	0.005	0.003	0.030	0.027	0.044	0.030	0.078
Saitozyma	0.000	0.000	0.035	0.021	0.006	0.000	0.000	0.000	0.000	0.000	0.064	0.000	0.032	0.000	0.023
Penicillium	0.027	0.000	0.004	0.007	0.044	0.047	0.000	0.000	0.031	0.000	0.000	0.002	0.000	0.000	0.000
Epicoccum	0.000	0.000	0.013	0.009	0.052	0.012	0.000	0.000	0.020	0.000	0.000	0.009	0.006	0.007	0.005
Phaeosphaeria	0.000	0.000	0.000	0.000	0.000	0.012	0.001	0.010	0.014	0.001	0.053	0.027	0.011	0.018	0.040
Schizothecium	0.012	0.000	0.000	0.000	0.000	0.000	0.000	0.000	0.000	0.000	0.064	0.034	0.011	0.041	0.020
Sterigmatomyces	0.000	0.003	0.000	0.066	0.023	0.000	0.000	0.000	0.000	0.000	0.000	0.008	0.000	0.004	0.000
Engyodontium	0.009	0.000	0.001	0.000	0.000	0.038	0.000	0.028	0.023	0.023	0.000	0.000	0.000	0.000	0.000
Periconia	0.006	0.005	0.018	0.006	0.019	0.008	0.000	0.000	0.000	0.000	0.006	0.001	0.010	0.013	0.009
Nigrospora	0.097	0.000	0.000	0.000	0.000	0.000	0.007	0.000	0.001	0.010	0.000	0.000	0.000	0.006	0.000
Mrakia	0.000	0.000	0.025	0.000	0.000	0.004	0.000	0.004	0.006	0.006	0.000	0.010	0.000	0.020	0.007
Others	0.249	0.114	0.154	0.276	0.160	0.144	0.049	0.119	0.155	0.092	0.265	0.204	0.151	0.331	0.256

Table S4 adjusted p-values for significantly different genera for each coral sample

genera	group1	group2	p	p.adj	p.signif
Apiotrichum	UT	BT	0.016	0.048	*
Botrytis	UT	BT	0.008	0.024	**
Botrytis	PBT	BT	0.008	0.024	**
Candida	UT	BT	0.008	0.024	**
Candida	PBT	BT	0.008	0.024	**
Curvularia	UT	BT	0.008	0.024	**
Curvularia	PBT	BT	0.032	0.063	*
Diatrypella	UT	PBT	0.008	0.024	**
Diatrypella	PBT	BT	0.008	0.024	**
Engyodontium	UT	PBT	0.032	0.063	*
Engyodontium	PBT	BT	0.008	0.024	**
Exserohilum	UT	PBT	0.032	0.063	*
Exserohilum	UT	BT	0.008	0.024	**
Golovinomyces	PBT	BT	0.008	0.024	**
Infundichalara	UT	BT	0.016	0.048	*
Infundichalara	PBT	BT	0.016	0.048	*
Mortierella	UT	BT	0.008	0.024	**
Mortierella	PBT	BT	0.008	0.024	**
Phaeosphaeria	UT	PBT	0.008	0.024	**
Phaeosphaeria	UT	BT	0.008	0.024	**
Phaeosphaeria	PBT	BT	0.032	0.032	*
Pithomyces	UT	PBT	0.016	0.048	*
Purpureocillium	UT	PBT	0.016	0.032	*
Purpureocillium	UT	BT	0.008	0.024	**
Saccharomyces	UT	PBT	0.008	0.024	**
Saccharomyces	UT	BT	0.008	0.024	**
Schizophyllum	UT	PBT	0.032	0.063	*
Schizophyllum	UT	BT	0.008	0.024	**
Schizothecium	UT	BT	0.016	0.032	*
Schizothecium	PBT	BT	0.008	0.024	**
Scleromitrula	UT	BT	0.016	0.048	*
Scleromitrula	PBT	BT	0.016	0.048	*
Setophaeosphaeria	UT	BT	0.016	0.048	*
Setophaeosphaeria	PBT	BT	0.016	0.048	*
Talaromyces	UT	PBT	0.016	0.048	*
Vishniacozyma	UT	BT	0.008	0.024	**
Vishniacozyma	PBT	BT	0.008	0.024	**

Table S5 Topological characteristics of the co-occurrence networks fungal interactions in different coral samples

Sample	Unbleached coral	Partly bleached coral	Bleached coral
Nodes	86	98	65
Edges	454	809	196
Positive correlations (%)	98.68%	93.82%	77.55%
Negative correlations (%)	1.32%	6.18%	22.45%
Average degree	10.558	16.51	6.031
Modularity	0.571	0.626	0.666
Average clustering coefficient	0.795	0.948	0.67
Average path distance	4.599	2.748	6.832
Density	0.124	0.17	0.094

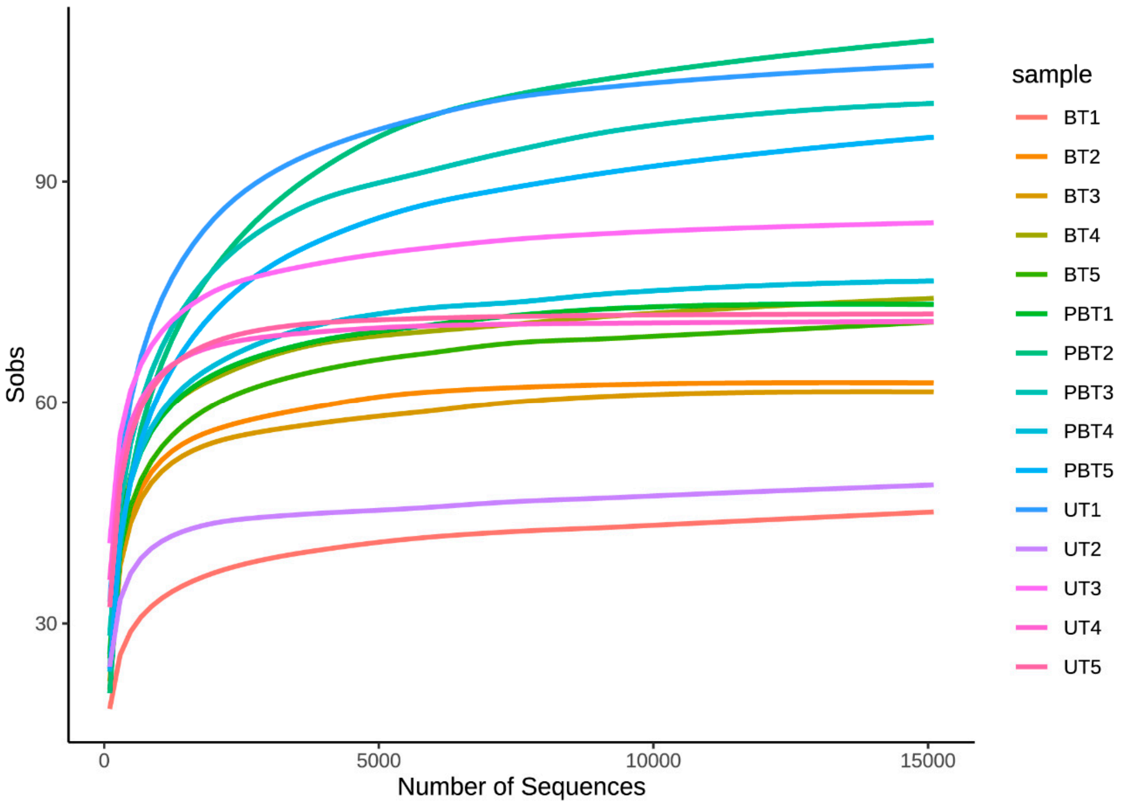

Figure S1 Rarefaction curves of the Sobs index for coral samples at different bleaching stages.
